# The acaricidal speed of kill of orally administered fluralaner against poultry red mites (*Dermanyssus gallinae*) on laying hens and its impact on mite reproduction

**DOI:** 10.1186/s13071-017-2534-5

**Published:** 2017-12-02

**Authors:** Maria D. Brauneis, Hartmut Zoller, Heike Williams, Eva Zschiesche, Anja R. Heckeroth

**Affiliations:** MSD Animal Health Innovation GmbH, Research Antiparasitics, Zur Propstei, 55270 Schwabenheim, Germany

**Keywords:** Poultry red mite, *Dermanyssus gallinae*, Laying hen, Fluralaner, Drinking water, Speed of kill, Reproduction

## Abstract

**Background:**

*Dermanyssus gallinae*, the poultry red mite, is a growing threat to chickens in poultry farms. This nocturnal hematophagous ectoparasite has a rapid rate of proliferation with a negative impact on the birds’ health, welfare and productivity resulting in severe economic consequences for poultry farmers. A study was performed with fluralaner, a novel systemic ectoparasiticide, to evaluate its effect on mite vitality and reproduction after oral administration to laying hens.

**Methods:**

Sixteen healthy hens were randomly allocated to two study groups (*n* = 8). One group was orally treated with fluralaner by gavage at a dose of 0.5 mg/kg bodyweight twice 7 days apart. The negative control group received no treatment. Hens in each group were repeatedly infested with approximately 200 unfed adult *D. gallinae* at 1, 5, 8, 12, 15, 19, 22 and 26 days after the initial administration. After infestation and feeding for 2.5 h, 25 engorged mites per hen were collected and incubated in tubes. Mites were assessed for vitality (dead/live) at 4, 8, 12, and 24 h after each infestation. Tubes containing eggs and/or living mites were incubated another 8 days for assessment of mite reproductive capacity.

**Results:**

Fluralaner demonstrated a fast speed of kill in mites within 4 h post-infestation for 12 days after treatment initiation. An efficacy (mite mortality) of 98.7–100% was achieved. At 15 days after treatment initiation, 100% efficacy was achieved within 24 h post-infestation, and no mite oviposition occurred during this period. Nineteen days after treatment initiation, the mites’ ability to generate nymphs was reduced by 90.8%, which decreased to < 24.1% at later infestations.

**Conclusions:**

Fluralaner administered orally to hens twice, 7 days apart, provides efficacy against experimental poultry red mite infestation for at least 2 weeks. The demonstrated rapid speed of kill results in substantial depletion of the mites’ oviposition and suggests that fluralaner can be an effective tool in the control of *D. gallinae*, one of the most urgent problems in poultry farms.

## Background

*Dermanyssus gallinae* (De Geer, 1778), the poultry red mite, is a growing threat to chickens in poultry farms. This blood-sucking ectoparasite especially affects hens in the egg-laying industry, but also hobby flocks [1, 2]. Estimates of poultry red mite prevalence suggest that up to 94% of commercial housing systems are infested [3]. *Dermanyssus gallinae* harms poultry either directly through blood-feeding or indirectly as a potential vector for numerous pathogens [4–6]. The poor health condition of mite infested poultry causes major economic losses for the egg industry (estimated around €230 million per year for Europe), due to diminished egg production and poor egg quality [7]. Moreover, mite-infested poultry houses can negatively impact farm workers’ health by exposing them to the parasite’s bite, an increasing risk for a zoonotic disease [8, 9]. In severe cases workers suffer amongst others from persistent skin irritation and itching dermatitis [1, 2, 10].

*Dermanyssus gallinae* is a ubiquitous hematophagous ectoparasite, preferably seeking its hosts at night, with almost all mite life stages, except for larvae, feeding on the blood of hens [1, 11]. After nocturnal feeding on hens for only 0.5 to 1.5 h mites retreat into cracks and crevices of nest boxes or the wider surroundings of the poultry house to digest their blood meal, mate and reproduce [12–14]. Female mites can feed and reproduce repeatedly, laying up to 30 eggs during their life time; the mite’s rapid life-cycle further contributes to the status of *D. gallinae* as a pest [1, 10]. Under optimal conditions, mite development from egg to adult takes around 7 days and includes 5 stages, i.e. eggs, larvae, protonymphs, deutonymphs and adults [1, 14]. Thus, a weekly doubling of the mite population is possible, and high parasite burdens can be reached (≥ 150,000 mites per bird are reported) [10, 12]. The impact of mite infestation on the birds’ health and well-being are serious: anemia through substantial blood loss, restlessness, feather picking, stress-caused aggression, weight loss and even death can occur. The production of blood-stained eggs and the reduction in egg size and quality are additional negative consequences for the farmers [1, 4, 13].

Because poultry red mites are resistant to desiccation they are able to survive without feeding in the absence of a host for up to 8 months [1]. Consequently, remaining mites can continuously multiply and build up a population over many production cycles, which often makes a one-time treatment of the poultry houses against *D. gallinae* challenging. Therefore, a fast, thorough and lasting elimination of the entire mite population is essential to effectively control this poultry pest. An acaricide with a fast mite killing effect which also interrupts the mites’ reproduction could achieve sustainable *D. gallinae* control in poultry farms.

Fluralaner, a member of the isoxazoline class, is a potent inhibitor of ligand-gated chloride channels with a high selectivity for the nervous system of acarids and insects [15–18]. Marketed for both, dogs and cats, fluralaner acts systemically against ticks and fleas [19]. Fluralaner also demonstrates the ability to considerably impact the reproduction of fleas, e.g. after oral administration to dogs [16, 17, 20, 21].

For poultry, a systemic medication via drinking water is a common and convenient treatment procedure and also a safe and simple method for *D. gallinae* control [22, 23]. This study was conducted to investigate fluralaner’s efficacy against poultry red mites including its speed of kill and possible effects on mite reproduction after oral administration to hens.

## Methods

### Study design

This study investigated the acaricidal efficacy of fluralaner against the poultry red mite, *D. gallinae*, including its speed of kill and impact on mite reproduction after oral administration to hens.

Sixteen laying hens were obtained from a commercial provider (Lohmann Selected Leghorn). During an acclimatization period they were randomly assigned to 2 study groups of 8 hens each (1 treatment group and 1 control group) based on their health status and body weight (range 960–1580 g). The hens were between 20 and 21 weeks old, and had not been exposed to any ectoparasite control product before start of the study. The hens were initially housed within their study group in pens enriched with nests, wooden shavings as nesting material, perches and cardboard. Due to a continuing social incompatibility among the hens, they were single-housed from day 13 after initial dose administration to the end of the animal phase. The housing room of hens was equipped with an artificial red light for 12 h per day and ambient temperature ventilation system.

All hens were clinically examined and weighed 1 day before the initial fluralaner dose administration (day 0) and at the end of the animal phase. Prior to the second administration (day 7), hens in the fluralaner group were weighed again for dose calculation purposes. The hens’ general health was monitored daily during the study.

### Treatment

The hens in the treatment group were administered fluralaner twice, and administrations were performed 7 days apart. Prior to both administrations, a stock solution containing 10 mg fluralaner/ml was diluted with tap water to obtain the intended dosing concentration of 0.2 mg fluralaner/ml. On day 0 and day 7, hens of the treatment group were orally administered at a dose of 0.5 mg fluralaner/kg body weight by gavage, and hens of the negative control group remained untreated. The administration volume per hen was calculated based on each hens’ body weight. Approximately 1, 6 and 24 h after the oral administration, hens were observed for possible adverse reactions, e.g. changes in general health condition and behavior.

### Mite infestation, collection and incubation

The poultry red mite (*D. gallinae*) isolate originated from a commercial layer farm in Germany and has been maintained as a colony at MSD Animal Health since 2001. The mite isolate is known to be susceptible to organophosphates, carbamates and spinosyns, but less susceptible to synthetic pyrethroids. All mites used for infestation showed natural mobility and were starved for 3 days prior to infestation. Six out of 8 hens in each study group were infested with approximately 200 unfed mites per infestation event on days 1, 5, 8, 12, 15, 19, 22 and 26 after the initial fluralaner administration. For an infestation period of 2.5 h, the hens were kept in separate polystyrene boxes (57 × 44 × 35 cm in size) and in the dark to enable mite feeding. The bottom of the box was outlined with a metal grid to prevent mites being damaged by the hen, and a mite-proof, but air-permeable lid ensured sufficient air flow during the infestation process.

At the end of an infestation period, hens were removed from the boxes and random aliquots of 25 (± 2) visibly engorged, adult mites were collected from each hens’ box by aspiration using a vacuum pump. Each mite aliquot was divided to 5 glass tubes of approximately 5 mites each. The glass tubes contained a colored paper strip to improve the visibility of mites in the tube thereby facilitating the monitoring of adult mites and their emerging juvenile stages. Mites in glass tubes were incubated in a climate chamber at 28 °C and 85% relative humidity, and were only temporarily removed from the chamber for the assessments of mite vitality and reproduction parameters. The incubation of mites was continued for 8 days to enable an emergence of nymphs.

### Assessment of adult mite vitality

Assessment of mite vitality was conducted repeatedly, i.e. at 4, 8, 12 and 24 h after a hen’s infestation had started, by counting the number of live/dead adult mites in a glass tube using a binocular (magnification 8- to 40-fold). The mites were classified as dead if they were in a dorsal position or no motility was detected.

### Assessment of mite reproduction

In conjunction with the mite vitality assessment conducted at 24 h post-infestation (see above), additional parasite counts were performed, including the numbers of live females/males, dead females/males, and viable eggs. Thereafter, daily counts of further developmental life stages were conducted during the incubation period, i.e. 8 days after an infestation, including the numbers of live females/males, dead females/males, viable eggs, live/dead larvae, and live/dead protonymphs.

### Data analysis

The descriptive data analysis was performed by means of Microsoft Excel® 2010 (Microsoft Corporation, Redmond, WA, USA) and statistical tests were performed using the software package SAS® (SAS Institute Inc., Cary, NC, USA, release 9.4). The treatment unit was the individual animal. The statistical unit was the individual mite for calculations of efficacy (mite mortality) and reproduction parameters.

### Calculation of acaricidal efficacy

The acaricidal efficacy was calculated as mite mortality (MM) at 4, 8, 12, and 24 h after each infestation, i.e. on days 1, 5, 8, 12, 15, 19, 22 and 26 post-administration. The following formula was used:

$$ \mathrm{MM}\ \left(\%\right)=\left({\mathrm{MR}}_{\mathrm{T}}\hbox{--} {\mathrm{MR}}_{\mathrm{C}}\right)/\left(1\hbox{--} {\mathrm{MR}}_{\mathrm{C}}\right)\times 100 $$

where MR is the adult mite mortality rate in the treatment group (MR_T_) and the control group (MR_C_).

MR was calculated for both study groups as follows:

$$ \mathrm{MR}\ \left(\%\right)=\mathrm{DM}/\mathrm{CM}\times 100 $$

where DM is the number of dead mites per group, and CM is the number of collected mites per group.

To confirm the validity of the efficacy results, adult mite mortality rates in both study groups were compared at each time point using Fisher’s exact test, one-sided with the level of significance set to α = 0.025.

### Calculation of effects on reproduction parameters

Treatment effects on the reproductive capacity of *D. gallinae* were calculated by quantitative comparison of the emerging juvenile stages (eggs, larvae, protonymphs) in the treatment to the control group. The reproduction parameters were calculated as (i) reduction of eggs per live female mite at 24 h post-infestation and at 8 days post-infestation; (ii) reduction of larval hatchability; (iii) reduction of nymphal conversion; and (iv) reduction of nymphal emergence. These calculations were only performed for infestations on days 19, 22 and 26 post-administration because no live mites or eggs were found in the treated group at earlier infestation events, i.e. day 1 to day 15 post-administration.

#### Reduction of eggs per live females (RELF)

RELF was calculated (a) 24 h post-infestation and (b) 8 days post-infestation as follows:

$$ \mathrm{RELF}\ \left(\%\right)=\left({\mathrm{ELF}}_{\mathrm{C}}\hbox{--} {\mathrm{ELF}}_{\mathrm{T}}\right)/{\mathrm{ELF}}_{\mathrm{T}}\times 100 $$

where ELF is the number of eggs per live female mite, in the treatment group (ELF_T_) and the control group (ELF_C_).

For (b), ELF was calculated daily (_d_) during the 8-day incubation period as the sum of newly observed eggs (EN_d_, calculation see below) divided by the sum of live females (LFM_d_):

$$ \mathrm{ELF}=\frac{\sum \limits_{\mathrm{d}=1}^8{\mathrm{EN}}_{\mathrm{d}}}{\sum \limits_{\mathrm{d}=1}^8{\mathrm{LFM}}_{\mathrm{d}}} $$

#### Reduction of larval hatchability (RLH)

RLH is an indicator for the egg viability and was calculated as follows:

$$ \mathrm{RLH}\ \left(\%\right)=\left({\mathrm{LH}}_{\mathrm{C}}\hbox{--} {\mathrm{LH}}_{\mathrm{T}}\right)/{\mathrm{LH}}_{\mathrm{T}}\times 100 $$

where LH is the larval hatch, in the treatment group (LH_T_) and in the control group (LH_C_).

LH contributes to subsequent molting of larvae and was calculated daily (_d_) during the 8-day incubation period as the sum of newly molted larvae (LH_d_) divided by the sum of newly observed eggs EN_d_:

$$ \%\mathrm{LH}=\frac{\sum \limits_{\mathrm{d}=2}^8{\mathrm{LH}}_{\mathrm{d}}}{\sum \limits_{\mathrm{d}=1}^8{\mathrm{EN}}_{\mathrm{d}}}\times 100 $$

EN_d_ was calculated as follows:

$$ {\mathrm{E}\mathrm{N}}_{\mathrm{d}}=\sum \limits_{\mathrm{T}=1}^{30}\left\{\begin{array}{c}{\mathrm{E}}_{\mathrm{T}\mathrm{d}}\\ {}{\mathrm{E}}_{\mathrm{T}\mathrm{d}}+{\mathrm{LH}}_{\mathrm{T}\mathrm{d}}-{\mathrm{E}}_{\mathrm{T}\left(\mathrm{d}\hbox{-} 1\right)}\\ {}0\end{array}\right.\kern1em {\displaystyle \begin{array}{l}\ \mathrm{if}\ \mathrm{d}=1\ \left(\mathrm{on}\ \mathrm{d}1\right)\\ {}\mathrm{if}\ \mathrm{d}>1\wedge {\mathrm{E}}_{\mathrm{T}\mathrm{d}}+{\mathrm{LH}}_{\mathrm{T}\mathrm{d}}\ge {\mathrm{E}}_{\mathrm{T}\left(\mathrm{d}\hbox{-} 1\right)}\\ {}\mathrm{else}\end{array}} $$

where E_Td_ is the number of eggs, and LH_Td_ is the number of newly molted larvae counted per tube (_T_) and day (_d_).

LH_d_ was calculated as follows:

$$ {\mathrm{L}\mathrm{H}}_{\mathrm{d}}=\sum \limits_{\mathrm{T}=1}^{30}\left\{\begin{array}{c}\left({\mathrm{L}}_{\mathrm{T}\mathrm{d}}\hbox{-} {\mathrm{L}}_{\mathrm{T}\left(\mathrm{d}\hbox{-} 1\right)}\right)+\left({\mathrm{N}}_{\mathrm{T}\mathrm{d}}\hbox{-} {\mathrm{N}}_{\mathrm{T}\left(\mathrm{d}\hbox{-} 1\right)}\right)\\ {}{\mathrm{L}}_{\mathrm{T}\mathrm{d}}\hbox{-} {\mathrm{L}}_{\mathrm{T}\left(\mathrm{d}\hbox{-} 1\right)}\\ {}0\end{array}\kern1em \right.{\displaystyle \begin{array}{l}\mathrm{if}\ {\mathrm{N}}_{\mathrm{T}\mathrm{d}}\hbox{-} {\mathrm{N}}_{\mathrm{T}\left(\mathrm{d}\hbox{-} 1\right)}\ge 0\wedge {\mathrm{N}}_{\mathrm{T}\mathrm{d}}-{\mathrm{N}}_{\mathrm{T}\left(\mathrm{d}\hbox{-} 1\right)}\ge {\mathrm{L}}_{\mathrm{T}\left(\mathrm{d}\hbox{-} 1\right)}\hbox{-} {\mathrm{L}}_{\mathrm{T}\mathrm{d}}\\ {}\mathrm{if}\ {\mathrm{N}}_{\mathrm{T}\mathrm{d}}\hbox{-} {\mathrm{N}}_{\mathrm{T}\left(\mathrm{d}\hbox{-} 1\right)}<0\kern0.5em \wedge \kern0.75em {\mathrm{L}}_{\mathrm{T}\mathrm{d}}-{\mathrm{L}}_{\mathrm{T}\left(\mathrm{d}\hbox{-} 1\right)}\ge 0\\ {}\mathrm{else}\end{array}} $$

where L_Td_ is the number of larvae, and N_Td_ the number of nymphs counted per tube (_T_) and day (_d_). The first day of incubation was excluded from the LH_d_ calculation (_d-1_, see formula above) because at this point in time a larval hatch from eggs is unlikely to occur.

#### Reduction of nymphal conversion (RNC)

RNC is an indicator for the reduction of larvae molting into nymphs and was calculated as follows:

$$ \mathrm{RNC}\ \left(\%\right)=\left({\mathrm{NCR}}_{\mathrm{C}}\hbox{--} {\mathrm{NCR}}_{\mathrm{T}}\right)/{\mathrm{NCR}}_{\mathrm{T}}\times 100 $$

where NCR is the nymphal conversion ratio, in the treatment group (NCR_T_) and the control group (NCR_C_).

NCR is an indicator for the magnitude of larvae developing into protonymphs and was calculated as follows:

$$ \mathrm{NCR}=\frac{\sum \limits_{\mathrm{d}=1}^8\sum \limits_{\mathrm{T}=1}^{30}{\mathrm{N}}_{\mathrm{T}\mathrm{d}}}{\sum \limits_{\mathrm{d}=1}^8{\mathrm{LH}}_{\mathrm{d}}}\times 100 $$

where N_Td_ is the number of nymphs per tube (_T_) and day (_d_); (see above for LH_d_ calculation).

#### Reduction of nymphal emergence (RNE)

RNE includes all other reproduction parameters (RELF, RLH, and RNC) and was calculated at the end of the 8-day incubation period as follows:

$$ \mathrm{RNE}\ \left(\%\right)=\left({\mathrm{NpF}}_{\mathrm{C}}\hbox{--} {\mathrm{NpF}}_{\mathrm{T}}\right)/{\mathrm{NpF}}_{\mathrm{C}}\times 100 $$

where NpF is the number of nymphs per female, in the treatment group (NpF_T_) and the control group (NpF_C_).

NpF was calculated as follows:

NpF = NE / FMI, where NE is the total number of nymphs emerged, and FMI is the total number of female mites incubated at each infestation.

## Results

### Acaricidal efficacy and speed of kill

**Fig. 1:**
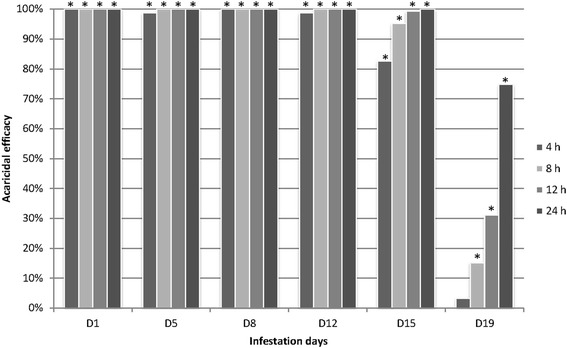
Acaricidal efficacy (%) after feeding of mites on fluralaner-treated hens. Assessment time points of the mites’ vitality: 4, 8, 12 and 24 h. * *P* < 0.0001

Figure 1 shows both, acaricidal efficacy against adult mites and speed of kill. For 12 days after the fluralaner administrations, efficacy was 98.7–100% at all assessment times, i.e. conducted at 4, 8, 12 and 24 h post-infestation. At 15 days, 82.6% efficacy was achieved at 4 h post-infestation that increased to 95.2% (8 h), 99.3% (12 h) and 100% at 24 h post-infestation. At 19 days efficacy was 3.3% (4 h), 15.2% (8 h), 31.1% (12 h) and 74.8% (24 h). Further acaricidal efficacy assessments were omitted, i.e. on days 22 and 26, because efficacies < 90% were observed for the infestation event on day 19 (Fig. 1).

A significant difference between study groups with *P* < 0.0001 was shown for each time point except for mite mortality at 4 h after the infestation on day 19 (efficacy 3.3%, *P* = 0.0292, Fisher’s exact test, one-sided).

### Mite reproduction parameters

For 15 days after fluralaner administrations, 100% of mites died within 24 h following the infestation and no oviposition occurred in the fluralaner-treated group. Thus, the reproduction parameters RELF, RLH, RNC, and RNE were derived from later infestations conducted on days 19, 22 and 26.

Reduction of eggs per live female mite (RELF) assessed at 24 h post-infestation was 82.2% (day 19), 3.6% (day 22) and 35.4% (day 26). RELF assessed 8 days post-infestation was 48.1% (day 19) and thereafter ≤ 15.9% (Table 1). Reduction of larval hatchability (RLH) was ≤ 2.6% (Table 2). Reduction of nymphal conversion (RNC) was ≤ 4.6% (Table 3). Reduction of nymphal emergence (RNE) was 90.8% (day 19) and thereafter ≤ 24.2% (Table 4).

**Table 1:** Reduction of eggs per live female after mite feeding on treated hens. Assessment 8 days post-infestation

Infestation day	ELF_C_	ELF_T_	RELF (%)
D19	0.8	0.4	48.1
D22	0.8	0.7	15.9
D26	0.8	0.7	10.6

*Abbreviations*: ELF, eggs per live female; _C_, control group; _T_, treatment group; RELF, reduction of eggs per live female mite

**Table 2:** Reduction of larval hatchability after mite feeding on treated hens

Infestation day	LH_C_ (%)	LH_T_ (%)	RLH (%)
D19	94.5	93.6	0.9
D22	91.6	90.5	1.2
D26	93.9	91.4	2.6

*Abbreviations*: LH, larval hatch; _C_, control group; _T_, treatment group; RLH, reduction of larval hatchability

**Table 3:** Reduction of nymphal conversion after mite feeding on treated hens

Infestation day	NCR_C_ (%)	NCR_T_ (%)	RNC (%)
D19	96.5	92.0	4.6
D22	92.4	92.0	0.4
D26	95.4	95.0	0.3

*Abbreviations*: NCR, nymphal conversion ratio; _C_, control group; _T_, treatment group; RNC, reduction of nymphal conversion

**Table 4:** Reduction of nymph emergence after mite feeding on treated hens

Infestation day	NpF_C_	NpF_T_	RNE (%)
D19	5.8	0.5	90.8
D22	5.0	3.8	24.2
D26	5.5	4.7	15.2

*Abbreviations*: NpF, nymphs per incubated female mite; _C,_ control group; _T_, treatment group; RNE, reduction of nymph emergence

**Fig. 2:**
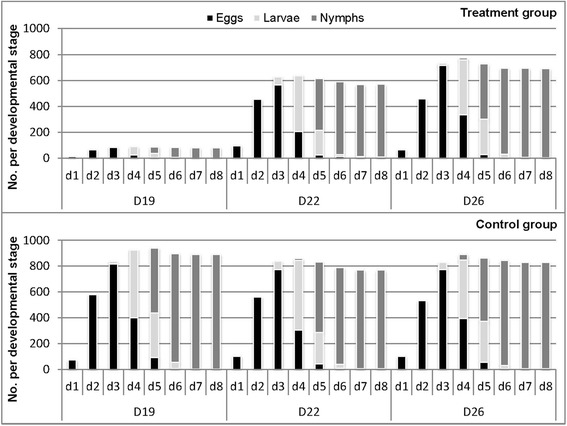
Composition of mite populations in treated vs control group. Infestation days: 19 (D19), 22 (D22) and 26 (D26). Incubation and assessment days: days 1 to 8 (d1-d8)

Figure 2 illustrates the composition of the mite populations in the treated group in comparison to that of the control group following the infestations on day 19, 22 and 26 during the 8-day incubation period. After the infestation on day 19, the mite population developing in the treated group was considerably smaller than in the control group. In both study groups the highest egg number was found on incubation day d3, the highest number of larvae on incubation day d4, and the highest number of nymphs emerged on incubation day d8 (Fig. 2).

No adverse reactions were observed in any of the study animals that were considered to be treatment related during the post-treatment observation period.

## Discussion

The preferred method for administering medical treatment in the poultry industry is orally via drinking water. So far this administration method has not been used for veterinary medicinal products in the treatment of the poultry red mite, *D. gallinae*. The current standard approach to control *D. gallinae* in poultry houses is a topically applied premise treatment by spraying of a pesticide, such as phoxim or spinosad, or dusting with products based on silicon dioxide; the latter can serve as an alternative to the use of targeted chemical acaricides [24–26]. Topical premise treatments have limitations regarding the extent of their antiparasitic effects, because the parasites’ hide-outs can be inaccessible with a spray or dust. As a result, some mites remain untreated and serve as a reservoir that is able to quickly build up a new bird-harming population in the stable [12, 27]. In this case, the labor-intense practice of topical premise treatment has to be performed more frequently [26]. Some premise treatments are approved to be performed in the presence of animals, and this can cause an additional disturbance of the birds, interrupting their normal behavior, and may temporarily impact food intake and productivity [24, 26]. Resistance development in poultry red mite to acaricides, such as organophosphates, can also play a role in the failure of mite treatment in poultry houses [13].

A systemic treatment approach that is able to prevent mite reproduction could help to overcome the current limitations in *D. gallinae* control, because any vital blood-feeding mite stage in a poultry house will inevitably feed on a treated bird, and become exposed to the systemic acaricide. Furthermore, an acaricidal effect that acts quickly enough would disrupt the mite’s egg production. The resulting beneficial effects are improved poultry health and well-being, along with a reduced potential of personnel being exposed to mite bites while working on poultry farms.

Oral administration of fluralaner is routinely used in dogs to provide a safe and fast acting antiparasitic treatment that also disrupts flea reproduction [17, 20]. Fluralaner’s proven acaricidal effects after oral treatment suggested that a similar approach would be effective for the treatment of *D. gallinae*. The recent approval of a product containing fluralaner for the treatment of poultry red mite by the European Medicine Agency confirms this conclusion (Exzolt™ authorization number: EU/2/17/212) [23, 28]. A 1% solution of fluralaner was orally administered to hens at the intended recommended dose regimen of 2 administrations of 0.5 mg fluralaner/kg body weight 7 days apart [29, 30]. Instead of offering the medication to birds via free access to drinking water, hens were administered by gavage to ensure that each hen received not more than the minimum intended dose for the efficacy assessments. The systemic fluralaner dose regimen was well tolerated by hens.

### Acaricidal efficacy and effects on mite reproduction

The study results confirm that oral administration of fluralaner to laying hens provides 99.3–100% acaricidal efficacy against *D. gallinae* infestation over a period of 15 days. Efficacy during this time frame was achieved within 12 h post-infestation preventing mites from laying any eggs. When efficacy assessments were conducted at 4 h post-infestation even this short period was sufficient to achieve high efficacy of 98.7–100% for a period of 12 days. The fast speed of kill (within 4–12 h) strongly contributes to the disruption of oviposition by poultry red mites, which generally deposit their eggs around 12 h after blood-feeding [1].

At 19 days after the initial administration to hens, the observed acaricidal efficacy of 74.8% did not reach common regulatory requirements [31, 32]. In the case that female mites managed to produce eggs, no ovicidal or larvicidal effects were observed but oviposition was considerably reduced by 50%, and the emergence of protonymphs was substantially reduced by 90.8% (day 19). This demonstrates that the intended dose regimen of fluralaner for the treatment of poultry red mites in laying hens prevents mite reproduction even after the initial acaricidal effect diminishes.

Under optimal environmental conditions for mites, which are common in commercial poultry houses, a period of 2 weeks allows *D. gallinae* to complete its life-cycle twice and therefore, mites are able to build up large mite populations within a short time [33]. The presented data indicate that 2 oral administrations of fluralaner, given to hens 7 days apart, has the potential to achieve a sustainable elimination of *D. gallinae* from poultry houses, by providing a potent acaricidal effect for at least 2 weeks that is achieved within 4–12 h, and disrupts the mite’s life-cycle.

## Conclusions

Oral administration of a systemically acting acaricide provides an alternative treatment option in the control of *D. gallinae* in poultry. Two oral fluralaner administrations given to laying hens 7 days apart provide efficacy against the poultry red mite for at least 2 weeks. The rapid mite-killing effect is achieved within 4–8 h after mite feeding, preventing mite oviposition and disrupting the mite’s life-cycle. Thus, oral administration of fluralaner can effectively treat existing poultry red mite infestation leading to the elimination of mites in poultry houses.

## Abbreviations

ELF: Eggs per live female
EN: Newly observed eggs
LFM: Sum of live females
LH: Larval hatch
MM: Mite mortality
MR: Mortality rate
NCR: Nymphal conversion ratio
NpF: Nymphs per incubated female
RELF: Reduction of eggs per live female
RLH: Reduction of larval hatchability
RNC: Reduction of nymphal conversion
RNE: Reduction of nymph emergence
T: Tube
